# Investigating the feasibility of cerebellar transcranial direct current stimulation to facilitate post-stroke overground gait performance in chronic stroke: a partial least-squares regression approach

**DOI:** 10.1186/s12984-021-00817-3

**Published:** 2021-01-28

**Authors:** Dhaval Solanki, Zeynab Rezaee, Anirban Dutta, Uttama Lahiri

**Affiliations:** 1 Electrical Engineering, Indian Institute of Technology Gandhinagar, Gujarat, India; 2 Biomedical Engineering, University at Buffalo SUNY, New York, USA

**Keywords:** Gait, Stroke, Cerebellum, Transcranial direct current stimulation

## Abstract

**Background:**

Investigation of lobule-specific electric field effects of cerebellar transcranial direct current stimulation (ctDCS) on overground gait performance has not been performed, so this study aimed to investigate the feasibility of two lobule-specific bilateral ctDCS montages to facilitate overground walking in chronic stroke.

**Methods:**

Ten chronic post-stroke male subjects participated in this repeated-measure single-blind crossover study, where we evaluated the single-session effects of two bilateral ctDCS montages that applied 2 mA via 3.14 cm^2^ disc electrodes for 15 min targeting (a) dentate nuclei (also, anterior and posterior lobes), and (b) lower-limb representations (lobules VIIb-IX). A two-sided Wilcoxon rank-sum test was performed at a 5% significance level on the percent normalized change measures in the overground gait performance. Partial least squares regression (PLSR) analysis was performed on the quantitative gait parameters as response variables to the mean lobular electric field strength as the predictors. Clinical assessments were performed with the Ten-Meter walk test (TMWT), Timed Up & Go (TUG), and the Berg Balance Scale based on minimal clinically important differences (MCID).

**Results:**

The ctDCS montage specific effect was found significant using a two-sided Wilcoxon rank-sum test at a 5% significance level for 'Step Time Affected Leg' (p = 0.0257) and '%Stance Time Unaffected Leg' (p = 0.0376). The changes in the quantitative gait parameters were found to be correlated to the mean electric field strength in the lobules based on PLSR analysis (*R*^2^ statistic = 0.6574). Here, the mean electric field strength at the cerebellar lobules, Vermis VIIIb, Ipsi-lesional IX, Vermis IX, Ipsi-lesional X, had the most loading and were positively related to the 'Step Time Affected Leg' and '%Stance Time Unaffected Leg,' and negatively related to the '%Swing Time Unaffected Leg,' '%Single Support Time Affected Leg.' Clinical assessments found similar improvement in the TMWT (MCID: 0.10 m/s), TUG (MCID: 8 s), and BBS score (MCID: 12.5 points) for both the ctDCS montages.

**Conclusion:**

Our feasibility study found an association between the lobular mean electric field strength and the changes in the quantitative gait parameters following a single ctDCS session in chronic stroke. Both the ctDCS montages improved the clinical outcome measures that should be investigated with a larger sample size for clinical validation.

*Trial registration:* Being retrospectively registered.

## Background

Stroke is a leading cause of disability across the globe, with 80.1 million (74.1 to 86.3) prevalent cases globally and 116.4 million (111.4 to 121.4) disability-adjusted life-years in 2016 [1]. Gait impairments occur in more than 80% of stroke survivors [2], which remain in 25% of all stroke survivors despite rehabilitation [3]. The recovery of independent walking requires considerable practice in stroke survivors [4] where neuroplasticity can be facilitated with adjuvant treatment with non-invasive brain stimulation (NIBS) techniques to the lower limb motor cortex of stroke survivors [5]. However, a recent systematic review and meta-analysis found only moderate-quality evidence that NIBS, including repetitive transcranial magnetic stimulation (rTMS) and transcranial direct current stimulation (tDCS), when combined with physical therapy, can be effective in improving post-stroke gait speed [6]. Another systematic review and meta-analysis [7] investigated NIBS, including rTMS and tDCS, in restoring functional balance and postural control in stroke survivors and found that the only rTMS had a significant effect. In the systematic review and meta-analysis [7], tDCS did not show significant therapeutic effects that may be due to inadequate dosing in the heterogeneous population. Individualized dosing of subthreshold stimulation is crucial in tDCS that involves passing constant weak direct current (generally of the order of 1–2 mA) via a pair of scalp electrodes (anodes and cathodes) to stimulate specific brain regions using electric field [8]. Here, tDCS effects on the brain tissue via electric field are governed by various parameters, including current intensity, electrode size, electrode placement, that may affect the efficacy of stimulation and its therapeutic outcomes [9]. Research studies on healthy humans have shown that the application of tDCS on the leg area of the motor cortex can increase the motor evoked potential in the lower limb muscles [10], which is a robust neurophysiological measure of the tDCS effect. However, the therapeutic effects of adjuvant treatment with tDCS during stroke rehabilitation can be challenging due to the heterogeneity in the residual brain state of the stroke survivors, leading to responders and non-responders in terms of their clinical outcomes. Therefore, a systematic analysis of the association of the electric field distribution in the brain tissue at the various targets of the motor network with its behavioral effects needs to be performed for stratification of stroke rehabilitation. Such systematic analysis is crucial to also identify non-responders to specific tDCS targets of the motor network, e.g., tDCS for the leg representations in the primary motor cortex at the interhemispheric fissure can be challenging, as demonstrated by Foerster et al. [11, 12], even in healthy humans.

Nevertheless, the application of tDCS at the primary and supplementary motor areas of the brain have been reported to affect the gait pattern of post-stroke patients [13, 14]. Specifically, Tahtis et al. [14] showed that bi-cephalic tDCS, with anode placed over the ipsilesional lower limb primary motor cortex and the cathode placed over the contra-lesional leg motor cortex, can bring clinical improvement in the gait functionality of post-stroke patients. In the other study, Manji et al. [13] applied tDCS with anode placed in the front of 'Cz' (10/20 EEG montage) and the cathode placed at the inion that improved gait speed (10-m walk test [15]) and walking ability (Timed-Up-and-Go [16]). Here, it is crucial to ensure that tDCS focally targeted the lower limb representations [11, 17], which can be ensured using computational head modeling, as demonstrated by Foerster et al.[11]. Moreover, therapeutic effects are also driven by the residual brain state of the stroke survivors; therefore, it is crucial to target various nodes of the motor network involved in motor learning during post-stroke rehabilitation due to the post-stroke heterogeneity of the residual brain state. The cerebellum and the motor cortex are known to be primarily involved in the adaptation and acquisition of locomotor behaviors [18]; where, the cerebellum is related to movement function, especially gait and balance, as well as error-based supervised learning [19] necessary in the early stages of motor learning. So, cerebellar tDCS (ctDCS) has been proposed to facilitate motor adaptation during a balance learning task [20]. Zandvliet and colleagues [20] found that contra-lesional anodal ctDCS improved the standing balance performance in a tandem stance position in chronic stroke survivors but not in age-matched healthy controls. Zandvliet and colleagues postulated that anodal ctDCS of the contra-lesional cerebellar hemisphere could strengthen the cerebellar-motor cortex (M1) connections to the affected cortical hemisphere. Zandvliet and colleagues also found that the ipsilesional anodal ctDCS did not improve standing balance performance; however, the neurophysiological reason remained unknown since the neurophysiological mechanisms, e.g., cerebellar brain inhibition (CBI), was not measured in that study. Also, Zandvliet and colleagues [20] did not present the lobular electric field distribution in the cerebellum, so the effects can be challenging to interpret without cerebellar lobule-specific dose information, especially in the elderly subjects [21].

In this feasibility study, we optimized cerebellar lobule-specific electric field distribution using our Cerebellar Lobules Optimal Stimulation (CLOS) pipeline [22] for deep ctDCS of the dentate nucleus (as well as both the lobes of the cerebellum) and the lower-limb representations (lobules VIIb-IX) in the cerebellum [23]. It is important to investigate the role of ctDCS in post-stroke gait rehabilitation [20] since "the motor cortex retains what the cerebellum learns [24]", i.e., unlike the primary motor cortex stimulation that may increase the retention of newly learned visuomotor skills, ctDCS may facilitate motor adaptation and early-stage error-based learning during repetitive balance training [24]. Here, the function of the cerebellum is 'to build internal models that predict the sensory outcome of motor commands and correct motor commands through internal feedback [25].’ Prior works have shown that the cerebellum aids visually-guided limb movement and facilitates learning of the limb movement trajectories [26, 27], which is crucial for post-stroke gait rehabilitation. Since the cerebellum also plays an important role in balance and coordination, which are critical to gait recovery, as evidenced by the TMS study [28], so it can be postulated that ctDCS may improve the gait and balance of post-stroke patients. In our prior work, offline deep ctDCS as a ‘priming’ intervention was found to facilitate standing balance function in chronic stroke survivors during a challenging functional reach task in virtual reality (VR) [23] using an adaptive balance training platform [29] for operant conditioning (with reward-based feedback) [30]. Also, our prior work [31] showed that anodal ctDCS of the anterior lobe of the cerebellum during visuomotor learning of myoelectric visual pursuit using electromyogram (EMG) from gastrocnemius muscle resulted in a statistically significant (p < 0.05) decrease in the reaction time post-intervention than baseline when compared to anodal ctDCS of the posterior lobe of the cerebellum as well as anodal ctDCS of combined anterior and posterior lobes of the cerebellum. Also, anodal ctDCS of combined anterior and posterior lobes of the cerebellum resulted in a significant decrease in the root mean square error post-intervention than in the baseline. Therefore, we found it crucial to investigate the behavioral effects of the lobular electric field distribution due to various ctDCS montages that are optimized using a computational pipeline [22].

In this feasibility study, we investigated two montages of ctDCS from our prior work [23] where our CLOS pipeline provided age-appropriate optimization of the ctDCS electrode montage [21] for bilateral deep ctDCS of the dentate nucleus and the lower-limb representations (lobules VIIb-IX) [23]. Specifically, we investigated the feasibility of a multivariate regression analysis [23] to associate the changes in the post-stroke overground gait performance (response variable) due to the lobular electric field distribution (predictor variable) for two optimized ctDCS electrode montages [21]. Based on our prior works [23], our goal was to target cerebellar regions related to dentate nuclei and the lower-limb representation (cerebellar lobules VIIb-IX), where the optimized electrode configuration for deep ctDCS of dentate nuclei also resulted in electric field distribution over combined anterior and posterior lobes of the cerebellum that was established in our prior work using computational modeling [23]. This is important since the dentate nucleus is involved in the planning, initiating, and modifying voluntary movements, e.g., in providing a learned timing signal required for motor preparation in the neocortex [32]. Here, the lobular electric field strength is proposed as a predictor of the changes in the gait parameters due to two different ctDCS interventions in terms of their electric field distribution [23] that was investigated in this study using multivariate regression analysis. In this feasibility study, the objectives were (i) evaluation of the acceptability of ctDCS by chronic stroke survivors based on a questionnaire, (ii) investigation of the effects of ctDCS on clinical gait and balance measures, as well as its multivariate regression analysis using the quantitative gait-related indices and the lobular electric field distribution, (iii) investigation of the differential effects of the two ctDCS montages on the clinical balance and gait measures, as well as quantitative gait-related indices including Step Length [33], Walk Ratio [34], Gait Stability Ratio [35], Symmetry Index [36], and other relevant gait parameters [37].

## Materials and methods

**Fig. 1:**
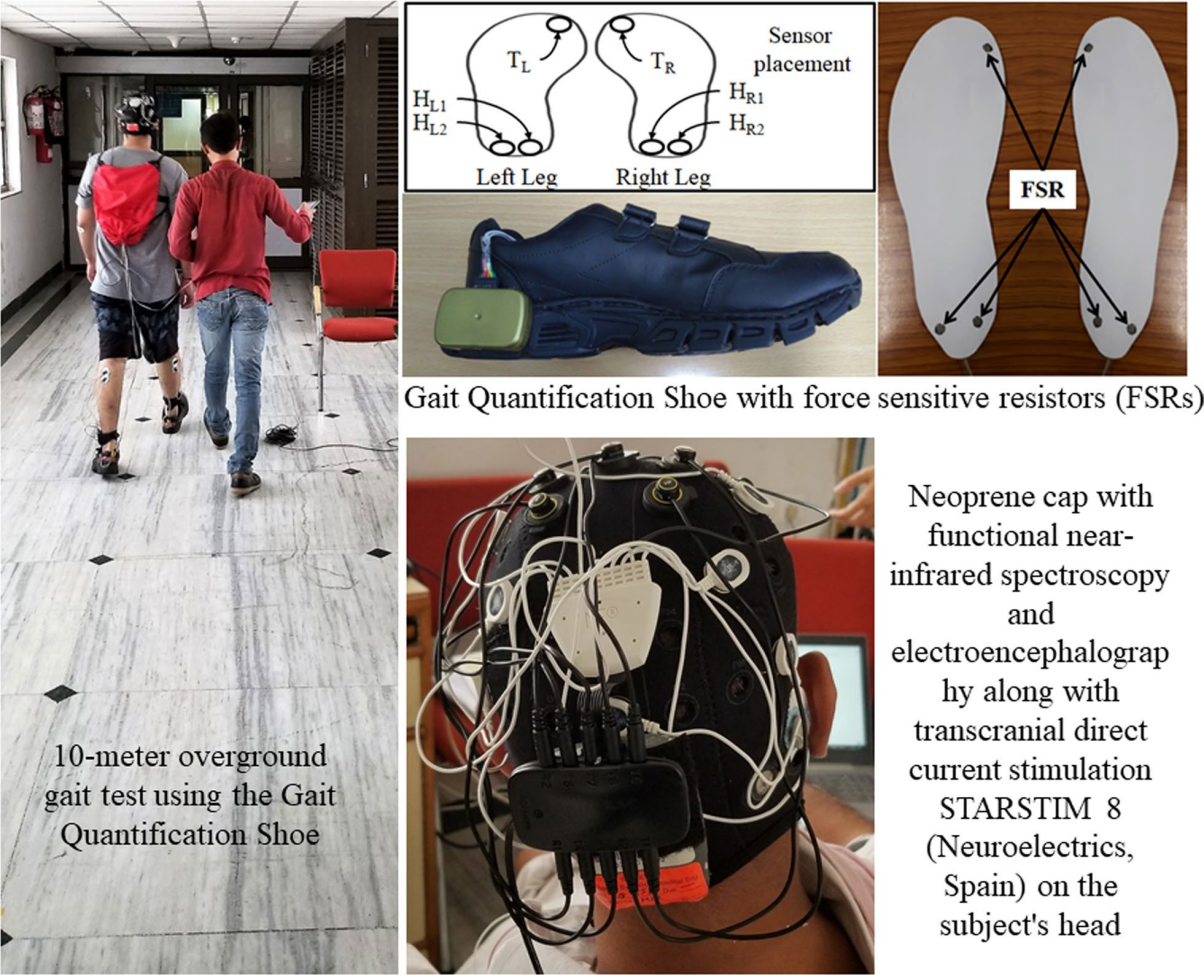
Experimental setup shown with a subject wearing the Gait Quantification Shoe, STARSTIM 8 stimulator with electrodes embedded in his cap, and walking on the 10-m walkway for the overground gait evaluation

**Fig. 2:**
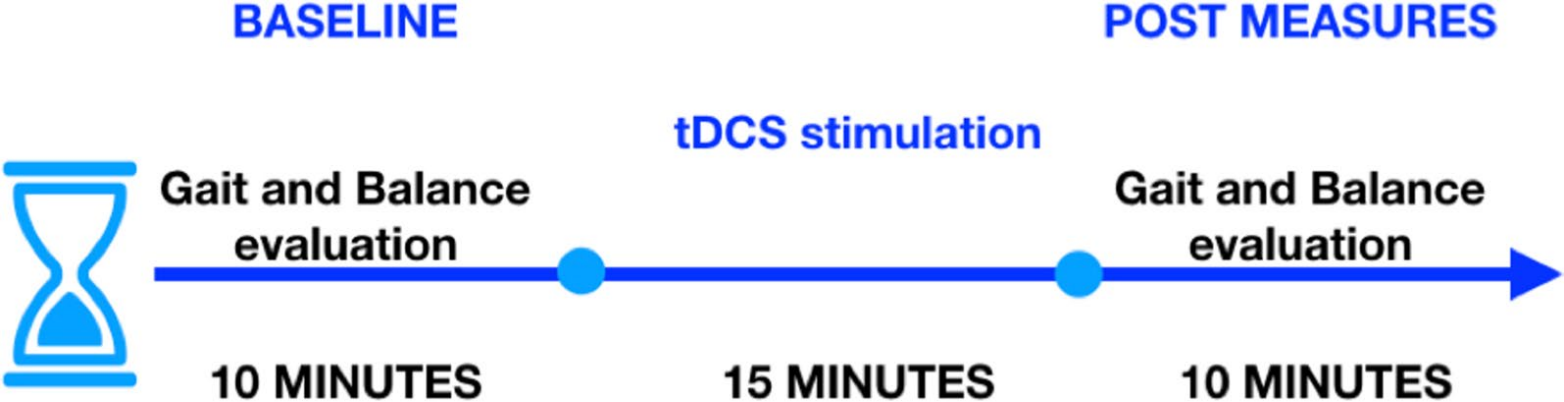
Block diagram for the experimental protocol

In this feasibility study at a low-resource point-of-care setting, we used our wearable gait quantification shoe [38] to quantify gait performance changes due to a single session of ctDCS in chronic stroke survivors. The experimental setup comprised of (i) the gait quantification shoe [38], and (ii) the wireless ctDCS cap with STARSTIM 8 stimulator (Neuroelectrics, Spain), as shown in Fig. 1. The gait quantification shoe characterized the gait parameters during overground walking in terms of Step Length [33], Walk Ratio [34], Gait Stability Ratio [35], Symmetry Index [36], and other relevant gait parameters [37], including Stride Time, Step Time, %Stance Time, %Swing Time, %Single Support Time, Cadence. Overground gait and balance evaluation were also performed based on the Ten-Meter walk test (TMWT) [39], Timed-Up-and-Go (TUG) [16], and Berg Balance Score (BBS) [40] before and after the ctDCS intervention, as shown in Fig. 2. A single session of ctDCS intervention was investigated based on its acute effects on the gait and balance measures from chronic (> 6 months’ post-stroke) hemiplegic patients.

### Study participants

The hemiplegic stroke survivors, who (i) were aged between 18 and 90 years, (ii) could walk independently for at least 10 m, (iii) could provide informed and written consent, and (iv) could understand instruction from the experimenter were contacted. Twelve post-stroke male subjects (P1-P12, Mean (SD) = 46(± 13) years) were selected for this study (see Table 1) from fourteen volunteers recruited by convenience sampling from collaborating hospitals. We selected chronic (> 6 months' post-stroke) stroke survivors with cerebral lesions but with an intact cerebellum (based on computerized tomography scan) so that the focal ctDCS electric field effects can be delivered to the cerebrum via the intact cerebellum [41]. Stroke survivors who underwent any recent surgery or were in the acute phase of stroke were excluded from the study. Written informed consent was obtained from each subject, and the multi-center research protocol for this study was approved by the All India Institute of Medical Sciences, New Delhi, India Institutional Review Board (IEC-129/07.04.2017), and Indian Institute of Technology Gandhinagar, India Institutional Review Board (IEC/2019–20/4/UL/046).

**Table 1:** Participant characteristics

Patient	Age group	Height	Weight	Post stroke period	Affected limb
ID	(years)	(cm)	(kilograms)	(years)	
P1	40–44	167	73	2	Right
P2	50–54	171	70	3	Right
P3	35–39	180	60	1	Left
P4	35–39	165	80	1	Right
P5	30–34	176	94	1	Right
P6	45–49	162	60	2	Left
P7	50–54	167	83	1	Left
P8*	70–74	163	76	6	Right
P9	40–44	164	60	3	Right
P10	45–49	167	70	2	Left
P11*	60–64	161	47	3	Right
P12	35–39	165	76	2	Right

*Subjects are dropouts

### Gait quantification shoe

In this study, we aimed to quantify gait-related indices by recording gait events using a pair of instrumented shoe [38]. Figure 1 shows the wearable device, namely the gait quantification shoes (Gait_Shoe_ henceforth) [38], that was used in this study to record the gait events. The Gait_Shoe_ consisted of insoles instrumented with force-sensitive resistors (FSRs) that were placed below the greater toe, lateral heel, and medial heel positions of each shoe to detect the gait events, e.g., heel-strike, toe-off, etc. These gait events were used to compute different gait-related indices, e.g., Step Length [33], Walk Ratio [34], Gait Stability Ratio [35], Symmetry Index [36], and other relevant gait parameters [37] including Stride Time, Step Time, %Stance Time, %Swing Time, %Single Support Time, Cadence. The Gait_Shoe_ transmitted the data wirelessly to a data logger computer for subsequent offline analysis.

- Computation of Step Length

Step Length is the distance between two successive contralateral heel-strikes during gait. We wanted to study the effects of ctDCS on Step Length since this is an essential indicator of the functional gait ability of hemiplegic post-stroke patients [33]. Here, we computed the average Step Length using the average Step Time (recorded by the Gait_Shoe_) and the average Walking Speed. The Step Time was measured by the Gait_Shoe_ from the time interval between two successive heel-strike events of the contralateral legs. Walking Speed (during the overground walk) was computed from the time taken to walk through a pre-defined distance. Subsequently, Step Length was calculated using Eq. (1). Finally, the Normalized Step Length was computed using the individualized height information [38] (Eq. (2)).

1 $${\text{Step Length}} = {\text{Step Time}}*{\text{Walking Speed}}$$

2 $${\text{Normalized Step Length}} = {\text{Step Length}}/{\text{Height}}$$

Normalized Step Length was computed separately for the affected and the unaffected sides of the hemiplegics.

b. Computation of gait stability ratio

The gait stability ratio (GSR) depends on Cadence (steps/s) and Walking Speed (m/s) [38]. The GSR is a good indicator of balance deficits in older adults [35]. The GSR takes into account the changes in Walking Speed that can influence the Step Length. Here, a decrease in GSR indicates increased double support time during walking. The GSR was computed using Eq. (3).

3 $${\text{Gait Stability Ratio}} = {\text{Cadence}}/{\text{Walking Speed}}$$

iii. Computation of walk ratio

The Walk Ratio (WR) [34] can describe a relation between Step Length and Cadence during walking. Importantly, WR is invariant during different speeds, uneven surface conditions but is affected by dual task-condition [42]. The Walk Ratio was computed using Eq. (4).

4 $${\text{Walk Ratio}} = {\text{Step Length}}/{\text{Cadence}}$$

Walk Ratio was computed separately for the affected and the unaffected sides of the hemiplegics.

iv. *Computation of Symmetry Index*

The Symmetry Index (SI) is a measure of the extent to which one makes symmetrical use of both the legs during walking [36]—the smaller the value of SI, the better is the gait symmetry. One of the distinctive characteristics of post-stroke gait is the impaired gait symmetry, particularly in hemiplegic patients [43]. Here, we computed the SI using the %stance phase (of a gait cycle) measured using the Gait_Shoe_ while considering the %stance for each of the left (X_L_) and right legs (X_R_). The SI was calculated using Eq. (5).

$${\text{SI}} = \left({\left( {{\text{X}}_{{\text{L}}} - {\text{X}}_{{\text{R}}} } \right)*{1}00)/(0.{5}*\left( {{\text{X}}_{{\text{L}}} + {\text{X}}_{{\text{R}}} } \right) } \right)$$ (5)

e. *Computation of Stride Time and Step Time*

The Stride Time, defined as the time interval between two successive heel-strike events of the ipsilateral legs [44], was computed using the Gait_Shoe_. The Stride Time and Step Time were computed separately for the affected and the unaffected sides of the hemiplegics.

f. *Computation of %Stance Time, %Swing Time*

The gait cycle can be considered broadly comprising of two main phases, namely Swing Phase and Stance Phase. A healthy gait cycle (GC) can be characterized by ~ 60% GC in the Stance Phase and ~ 40% GC in Swing Phase [44]. The Stance Phase can be defined as the phase in which the foot stays in contact with the base of support (e.g., the floor) during the gait cycle, while the Swing Phase can be defined as the phase in which the foot is not in contact with the base of support [44]. In this study, the Stance Time was computed as the time interval between the successive heel-strike and toe-off events of the ipsilateral leg based on the foot contact with the floor. The %Stance time was computed by evaluating the Stance Time as a percentage of the gait cycle time. Similarly, the Swing Time was computed as the time interval between the successive toe-off and heel-strike events of the ipsilateral leg when the foot was not in contact with the floor. The %Swing Time was computed by evaluating the Swing Time as a percentage of the gait cycle time. The %Stance Time and %Swing Time were computed separately for the affected and the unaffected sides of the hemiplegics.

g. *Computation of %Single Support Time*

The Single Support Time (SST) is the duration of a gait cycle for which only one foot stays in contact with the base of support (such as the floor) while supporting the entire weight of the body on that leg under dynamic stability during a gait cycle which is important for fast walking [45]. The SST can be computed as the swing time of the contralateral leg. Gait cycle duration was measured using the time interval between the two consecutive heel-strike events of the same leg. Subsequently, the %SST was calculated for each leg using Eq. (6). The %SST was computed separately for the affected and the unaffected sides of the hemiplegics.

$$\%SST = \frac{Swing Tim{e}_{CL}*100}{Gait Cycle Time}$$ (6)

h. *Computation of Cadence*

Cadence can be defined as the number of steps walked per minute [37]. The Cadence was computed as the number of heel-strike events registered per minute, considering both the affected and the unaffected legs.

### Optimization of the electrode montage (age-specific computational modeling of ctDCS)

We used age-specific MRI templates that were obtained online at https://jerlab.sc.edu/projects/neurodevelopmental-mri-database/ with the permission of Dr. John Richards. The data comprised of average T1-weighted MRI with the segmentation priors for the gray matter (GM), white matter (WM), and cerebrospinal fluid (CSF). For this, we chose the age group that matched the age of our subjects for this study. A Realistic volumetric Approach to Simulate Transcranial Electric Stimulation (ROAST) [46] was used to create a tetrahedral volume mesh of the head. ROAST used SPM12 ("SPM—Statistical Parametric Mapping") to segment the head and brain. After segmentation, five tissues were identified for the tetrahedral volume mesh, namely, Scalp, Skull, Cerebrospinal Fluid (CSF), Gray Matter (GM), and White Matter (WM). These different brain tissues for the volume mesh were modeled as different volume conductors for Finite Element Analysis (FEA) in the ROAST. Here, isotropic conductivity used for the different brain tissues [46] were (in S/m): Scalp = 0.465; Skull = 0.01; CSF = 1.654; GM = 0.276; WM = 0.126. For further details on the head modeling, please refer to our prior works [22, 23].

The Electric Field (EF) distribution was found for two different ctDCS montages based on the subject's age-specific head model that was created from MRI templates (https://jerlab.sc.edu/projects/neurodevelopmental-mri-database/). The boundary condition was set as 2 mA injection current (Neumann boundary condition) with the following electrode configurations from our prior work where we performed optimization of the electrode montage [23]:

- *Optimized configuration for dentate nuclei stimulation *[23]: 3.14 cm^2^ disc anode was PO10h (10/5 EEG system), and 3.14 cm^2^ disc cathode was placed at PO9h (10/5 EEG system) for ctDCS with 2 mA direct current.
- *Optimized configuration for leg lobules VII-IX stimulation* [23]: 3.14 cm^2^ disc anode was Exx8 (electrodes defined by ROAST using "unambiguously illustrated (UI) 10/5 system" [47]), and 3.14 cm^2^ disc cathode was placed at Exx7 (defined by ROAST) for ctDCS with 2 mA direct current.

In all the simulations, the voxel size was considered as 1mm^3^. The contra-lesional anode and ipsilesional cathode injected the specified amount of current in the volume conductor, i.e., the head model. Finite Element Analysis (FEA) was conducted on each age-specific head model to compute the ctDCS induced EF in the brain tissues. The electric field was computed at all the voxels (voxel size 1 mm^3^) of the cerebellar lobules that were normalized for flatmap using a spatially unbiased atlas for the cerebellum and brainstem (SUIT) [48]. Here, the cerebellar lobular electric field distribution was found as flatmap using SUIT [48] and T1-weighted images that were fitted to the SUIT template of the human cerebellum in SPM12 ("SPM—Statistical Parametric Mapping": https://www.fil.ion.ucl.ac.uk/spm/software/spm12/). The cerebellar mask was visually checked in MRIcron, and the non-linear deformation was then applied to each EF image. The volume of the cerebellar lobules, defined by the SUIT atlas [48], was used for the extraction of the lobular EF distribution. Also, we customized SUIT codes to extract the EF distribution at the left and the right dentate nucleus.

### Experimental setup and data analysis

Figure 1 shows the experimental setup for the clinical study in a low-resource point-of-care setting with a subject walking on the 10-m walkway for overground gait evaluation. The study required a commitment of about 30 min from each participant.

- a. *Cerebellar tDCS intervention*

Based on our prior work [23], 15 min of 2 mA bilateral ctDCS was delivered in a repeated measure single-blind crossover design using two bipolar montages with a circular (1 cm radius) contra-lesional anode. The two bipolar montages were allocated in random order with 2–3 days’ washout period between the ctDCS sessions, and the subjects were blinded to the montage by keeping all the four stimulation electrodes (two anodes and two cathodes for two ctDCS montages) always embedded in their cap. The electrode locations in the cap were based on the ROAST toolbox [46], and "unambiguously illustrated (UI) 10/5 system" [47]; 1. PO9h–PO10h, and 2. Exx7–Exx8. The experimental setup is shown in Fig. 1 (see the right bottom inset with the neoprene cap), and the experimental protocol is shown in Fig. 2, where overground quantitative gait, as well as clinical gait (TMWT [39]) and balance evaluations (TUG, BBS), were performed before and after the ctDCS intervention to compute a percent normalized change measure, $\frac{100}{(\mathrm{POST}+\mathrm{PRE})}$(POST–PRE).

- b. Experimental setup for overground gait analysis

The experimental setup for the overground gait analysis consisted of (i) 10 m long straight overground pathway (for TMWT [39]) marked with start and end lines, (ii) data-logger computer, and (iii) a pair of Gait_Shoes_. We investigated the effects of ctDCS on gait characteristics during the 10 m overground walk – see Fig. 1. Once the participant arrived at the study hall, they were asked to sit and relax for about 5 min. Then, the experimenter explained to the participant what he was expected to do in the study as well as the risks. After informed consent, the baseline clinical measures were recorded. Then, the experimenter helped the participant to wear the Gait_Shoe_ [38]_._ Subsequently, the experimenter prepared the participant for ctDCS by placing the neoprene cap combined with a battery-driven wireless stimulator, STARSTIM8 (Neuroelectrics, Spain), and the gel-based electrodes. The participants were informed that they could discontinue the study in case of any discomfort.

Once the participant was ready to start the study, they were asked to walk on a 10 m long straight path (overground) marked with a start and stop lines at their self-selected comfortable speed, and the participant's overground Walking Speed (Speed_OG_) was computed. After this, the participant was asked to sit and relax on a chair for about 5 min. Subsequently, ctDCS was administered using one of the two ctDCS montages for 15 min at the rest condition with a dosage of 2 mA [23]. Following this, the participant repeated the 10 m overground walk, followed by an assessment of the clinical gait and balance measures (TMWT, TUG, and BBS). The gait performance of the post-stroke participants was also quantified using Gait_Shoe_ in terms of the gait-related indices, as described earlier. Therefore, the post-stroke participants performed two trials of the overground walk, pre, and post ctDCS intervention, at their self-selected walking speed while wearing the Gait_Shoe_, as illustrated in Fig. 2. We also evaluated the acceptability of the ctDCS intervention in post-stroke subjects based on a questionnaire (see Supplementary Materials) where we collected subjective feedback from the post-stroke participants prior to (*Pre*_*tDCS*_), during (*Active*_*tDCS*_), and post (*Post*_*tDCS*_) application of ctDCS.

- c) Statistical analysis and the partial least squares regression

A two-sided Wilcoxon rank-sum test was performed at the 5% significance level on the percent normalized change measures,$\left(\mathrm{POST}-\mathrm{PRE}\right)\frac{100}{(\mathrm{POST}+\mathrm{PRE})}$ for the null hypothesis that the two ctDCS montages led to the same percent normalized change in the quantitative gait parameters from the same continuous distributions with equal medians. Multivariate regression analysis was conducted to relate the changes in the balance and gait measures to the lobular electric field distribution due to ctDCS montages. Here, multicollinearity can occur when independent variables (predictors) are correlated. In our prior work [23], we have presented principal component regression analysis for multivariate linear regression of the lobular electric field distribution as the predictor with the behavioral outcomes as the response variables. The goal is to extract the relation between electric field distribution and the behavioral effects of ctDCS where Partial Least Squares (PLS) can be a promising multivariate statistical technique that can combine the information about the variances of both the predictors and the responses while also considering the correlations among them [49]. In this study, we applied a PLS regression (PLSR) approach to analyze the associations between the lobular electric field distribution as the predictor with the gait outcome measures as the response variables. Although statistical inference is the strength of the PLSR approach using computational cross-validation methods (e.g., jackknife, bootstrap) [49]; however, we will apply PLS as a correlation technique in this study. The matrix of correlations between the lobular electric field distribution as the predictor with the gait outcome measures as the response variables is subjected to the singular value decomposition that results in the singular vectors called saliences. The lobular electric field distribution as the predictor with the gait outcome measures as the response variables can be projected onto their respective saliences, which creates latent variables that are linear combinations of the original variables. Here, PLS searches for latent variables that express the largest amount of information common to both the lobular electric field distribution as the predictor and the gait outcome measures as the response variables. This is a fixed-effect model where the results can only be interpreted with respect to the current data sets from this study. In this study, PLS analysis was performed on the percent normalized change measures, $\left(\mathrm{POST}-\mathrm{PRE}\right)\frac{100}{(\mathrm{POST}+\mathrm{PRE})}$ of gait indices from the Gait_shoe_ as the response variable, where the lobular electric field distribution for both the montages across all the subjects (found after centering the data and then singular value decomposition) was the predictor.

## Results

### Acceptability of ctDCS

Once the cap with the electrodes and the portable tDCS device was placed on the participant's head, we obtained *Pre*_*tDCS*_ feedback from the participant to understand whether they were comfortable with the neoprene cap. Two subjects, P8 and P11, had challenges with the fitting of the ctDCS cap and the gel electrodes on the scalp, so they left the study. The rest of the 10 participants expressed that they were comfortable wearing the ctDCS cap with gel electrodes. The *Pre*_*tDCS*_ baseline feedback was followed by the feedback after the administration of the ctDCS, i.e., the *Active*_*tDCS*_. Except for two subjects who left the study at the baseline (*Pre*_*tDCS*_) stage, none of the ten participants expressed any discomfort with the neoprene cap or the ctDCS intervention (at *Active*_*tDCS*_). The ten participants reported a tolerable tingling sensation on the scalp for the first few seconds. After the application of ctDCS, the *Post*_*tDCS*_ verbal feedback revealed that none of the participants had any adverse effects, such as a sensation of tissue burning, nausea, headache, etc. (questionnaire provided in the supplementary materials). Also, no skin reddening (at the location of electrodes) of the scalp was noticed during a visual inspection.

### Effects of ctDCS on Gait-related Indices measured using the Gait_Shoe_

**Fig. 3:**
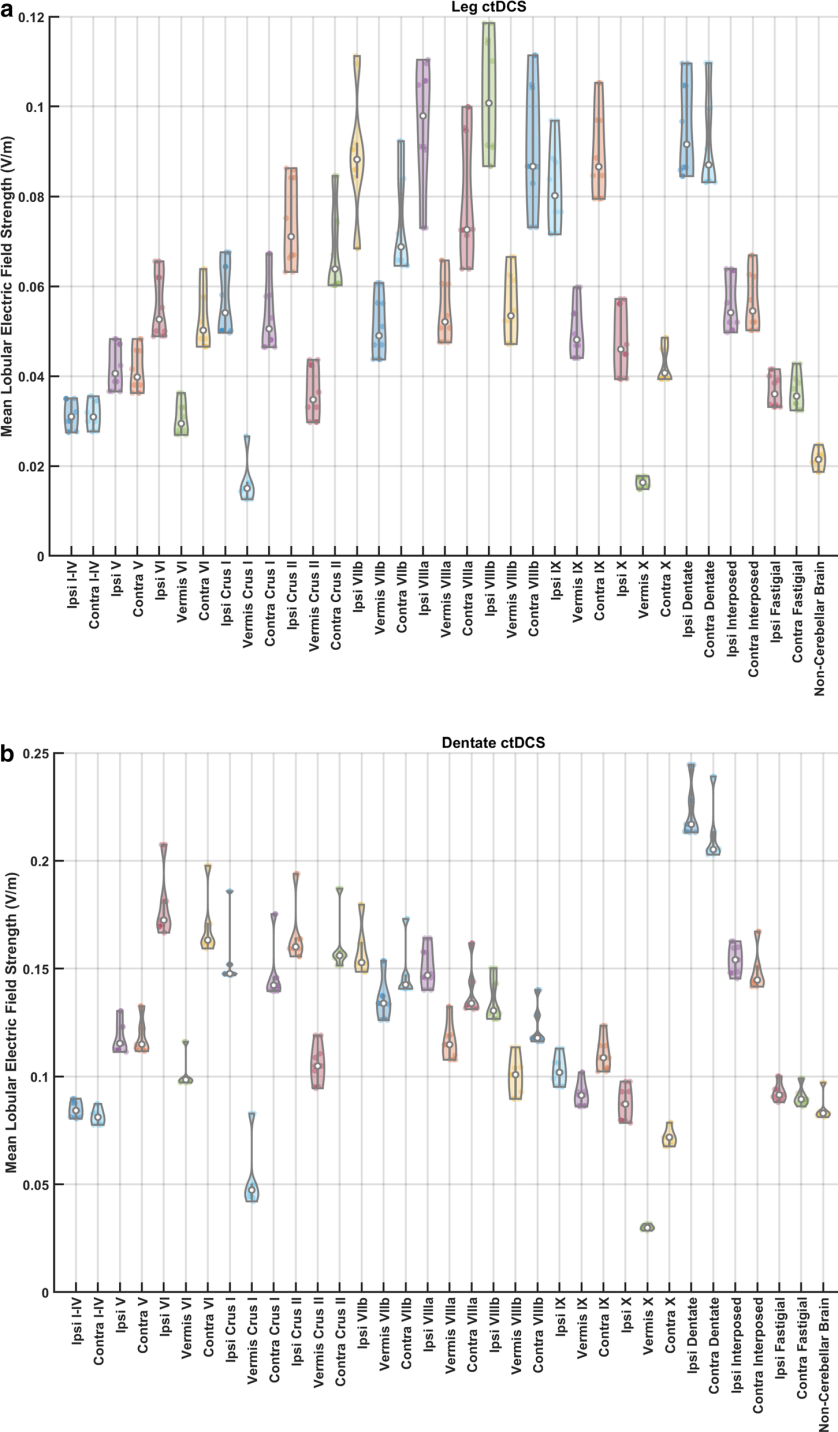
**a** Violin plot of the mean lobular electric field strength (V/m) across 10 participants for the optimized configuration for leg lobules VIIb-IX stimulation. **b** Violin plot of the mean lobular electric field strength (V/m) across 10 participants for the optimized configuration for dentate nuclei stimulation. ‘Contra’ is contra-lesional and ‘Ipsi’ is ipsi-lesional. Violin plot allowed visualization of the distribution of the data and its probability density where the box plot (with median, interquartile range, upper adjacent value, lower adjacent value) is combined with the probability density placed on each side

Effects of ctDCS on post-stroke overground gait were quantified using the Gait_Shoe_ that measured gait-related indices, e.g., Step Length, Gait Stability Ratio, Walk Ratio, and Symmetry Index. Also, the mean lobular electric field strengths for all the 10 participants using their age-specific head model were found for both the ctDCS montages, dentate ctDCS, and leg (lobules VIIb-IX) ctDCS, as shown in Fig. 3. Figure 3a shows that the leg ctDCS, while targeting the mean electric field strength at the posterior cerebellar lobules VIIb-IX (> 0.08 V/m), also affected the dentate nuclei at a comparable electric field strength (0.1 V/m). Figure 3b shows that the dentate ctDCS affected the dentate nuclei at greater than 0.2 V/m mean electric field strength in addition to anterior and posterior lobes of the cerebellum (> 0.1 V/m). These results are based on computational modeling using the subject's age-matched healthy MRI templates (four post-stroke subjects were left hemiplegic and the remaining six right hemiplegics as shown in Table 1) that showed leg ctDCS affected the dentate nuclei as well as the posterior cerebellar lobules VIIb-IX (> 0.08 V/m). In contrast, the dentate ctDCS affected the dentate nuclei (> 0.2 V/m) as well as the anterior and posterior lobes of the cerebellum (> 0.1 V/m).

**Fig. 4:**
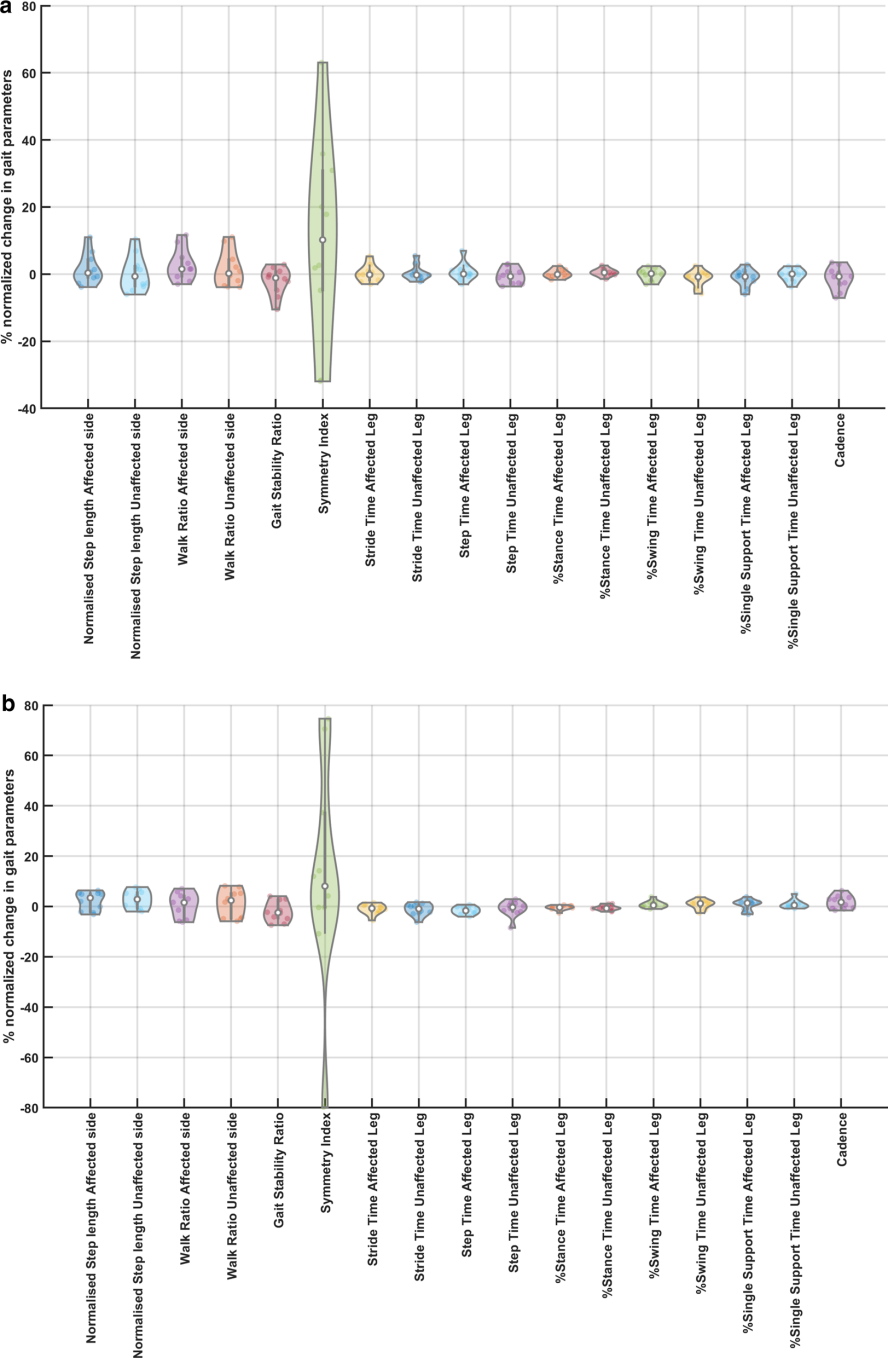
**a** Violin plot of the mean % change in the gait parameters across 10 participants due to ctDCS optimized for leg lobules VIIb-IX stimulation. **b** Violin plot of the mean % change in the gait parameters across 10 participants due to ctDCS optimized for dentate stimulation.Violin plot allowed visualization of the distribution of the data and its probability density where the box plot (with median, interquartile range, upper adjacent value, lower adjacent value) is combined with the probability density placed on each side

Figure 4 shows the percent normalized change measures, $\left(\mathrm{POST}-\mathrm{PRE}\right)\frac{100}{(\mathrm{POST}+\mathrm{PRE})}$, in the gait parameters across the 10 participants due to the two ctDCS montages. The distribution shown in the violin plots in Fig. 4 was found to be mostly non-Gaussian. Therefore, a non-parametric two-sided Wilcoxon rank-sum test at a 5% significance level was used to find the difference in the effects between the two ctDCS montages where statistically significant effect was found for 'Step Time Affected Leg' (p = 0.0257) and '%Stance Time Unaffected Leg' (p = 0.0376). Dentate ctDCS decreased the 'Step Time Affected Leg' and the '%Stance Time Unaffected Leg' while the leg (lobules VIIb-IX) ctDCS increased them both. This resulted in an increase in the 'Cadence' by dentate ctDCS and a decrease by leg (lobules VIIb-IX) ctDCS; however, this effect was found to be insignificant (p = 0.0890). Also, the montage specific effect was found to be insignificant for 'Normalised Step length Affected side' (p = 0.6776), 'Normalised Step length Unaffected side' (p = 0.1859), 'Walk Ratio Affected side' (p = 0.5205), 'Walk Ratio Unaffected side' (p = 0.7337), 'Gait Stability Ratio' (p = 0.7913), 'Symmetry Index' (p = 0.9097), 'Stride Time Affected Leg' (p = 0.4727), 'Stride Time Unaffected Leg' (p = 0.3847), 'Step Time Unaffected Leg' (p = 0.7913), '%Stance Time Affected Leg' (p = 0.4274), '%Swing Time Affected Leg' (p = 0.5205), '%Swing Time Unaffected Leg' (p = 0.0539), '%Single Support Time Affected Leg' (p = 0.1212), '%Single Support Time Unaffected Leg' (p = 0.3075).

**Fig. 5:**
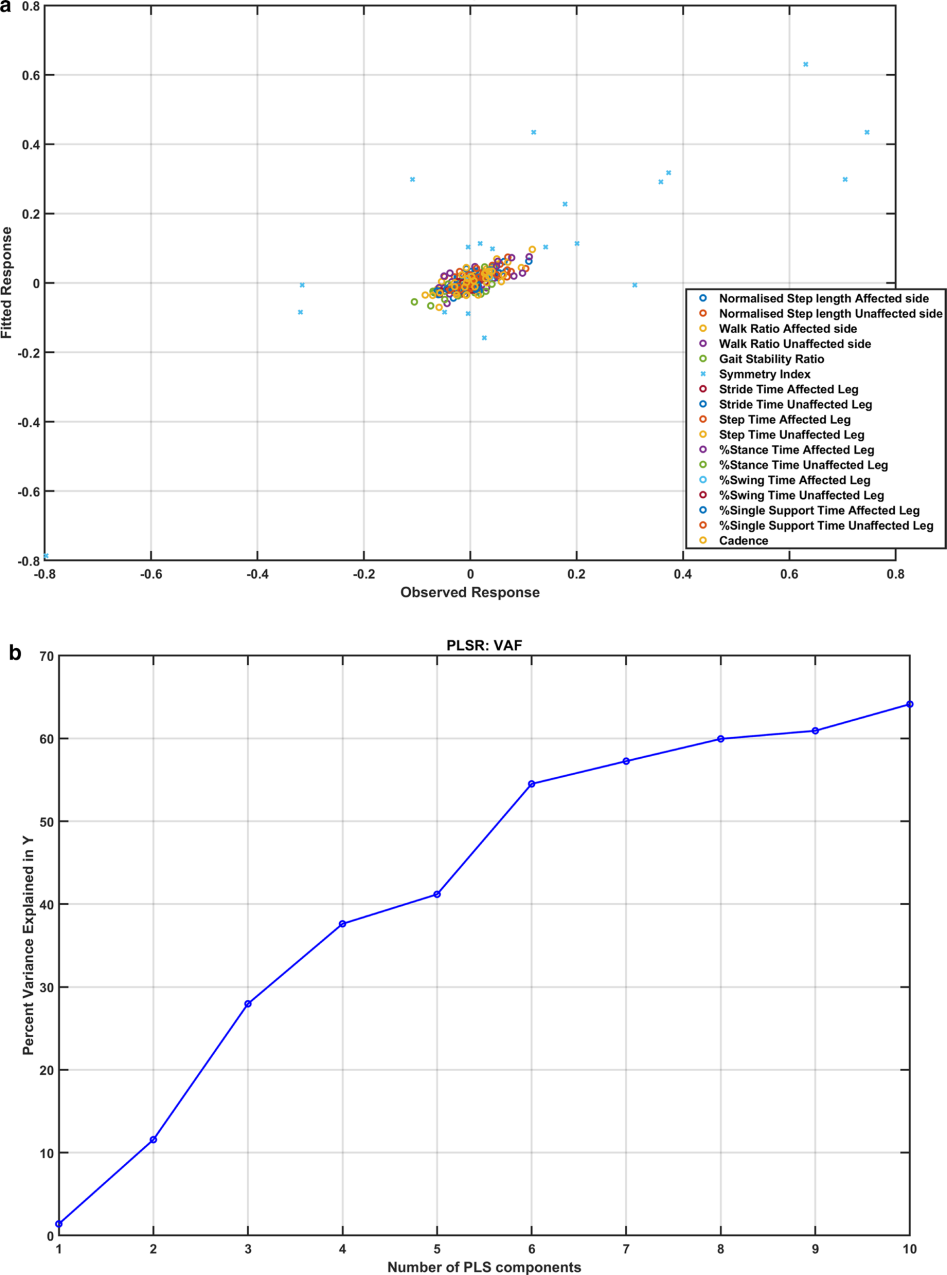
**a** The scatter plot shows a reasonable correlation between fitted and observed responses after partial least squares (PLS) regression for all the response variables (percent normalized change in gait parameters). **b** Greater than 60% of the variance in the response variables (percent normalized change in gait parameters) was explained by the first ten components of the predictor variables (mean lobular electric field strength)

The effects of the two montages of the ctDCS were found similar across ten subjects during clinical assessments (details in Additional file 1). Figure 5a shows a reasonable correlation between fitted and observed responses using PLS analysis with the mean lobular electric field strength as the predictors confirmed by the *R*^2^ statistic = 0.6574. Residuals passed the Lilliefors test for two-sided goodness-of-fit for normality. Choosing the number of components in a PLS regression (PLSR) model is a critical step where greater than 60% of the variance in the response variables (percent normalized change in gait parameters) was explained by the first ten components of the predictor variables (mean lobular electric field strength), as shown in Fig. 5b.

**Fig. 6:**
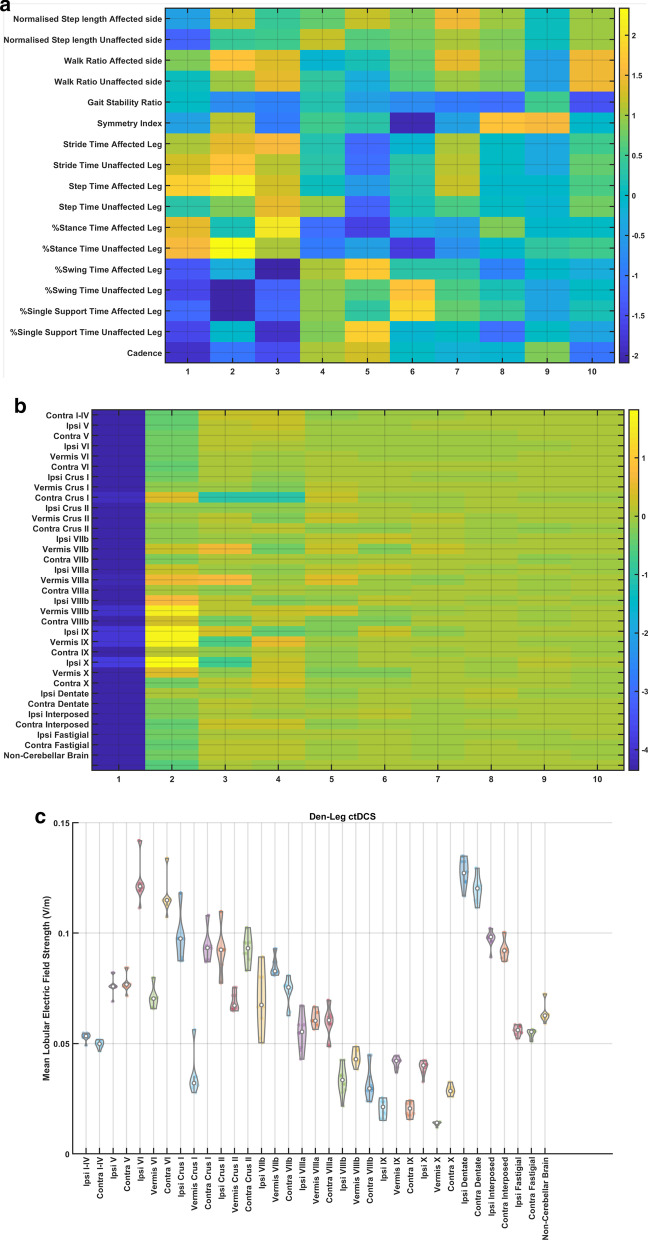
Partial least squares (PLS) component loadings. **a** loadings of the latent variables of the response variables (percent normalized change in gait parameters) where the components are in the x-axis. **b** loadings of the latent variables of the predictor variables (mean lobular electric field strength) where the components are in the x-axis. **c** contrast in mean lobular electric field strength (V/m) for 10 participants between the dentate and the leg lobules VII-IX ctDCS montages. ‘Contra’ is contra-lesional and ‘Ipsi’ is ipsi-lesional

Furthermore, the loadings of the latent variables on the response variables (percent normalized change in gait parameters) and the predictor variables (mean lobular electric field strength) are shown in Fig. 6a b, respectively. Here, we found that the mean lobular electric field strength in the cerebellar lobules, Vermis VIIIb, Ipsi-lesional IX, Vermis IX, Ipsi-lesional X, were positively related by the PLSR component 2 to the 'Step Time Affected Leg' (p = 0.0257) and '%Stance Time Unaffected Leg' (p = 0.0376), that showed significant effects between the two ctDCS montages based on two-sided Wilcoxon rank-sum test. Also, the mean lobular electric field strength in the cerebellar lobules, Vermis VIIIb, Ipsi-lesional IX, Vermis IX, Ipsi-lesional X, were negatively related by the PLSR component 2 to the '%Swing Time Unaffected Leg' (p = 0.0539), '%Single Support Time Affected Leg' (p = 0.1212). Figure 6c shows that the cerebellar lobules, Vermis VIIIb, Ipsi-lesional IX, Vermis IX, Ipsi-lesional X, had the lowest difference in their mean lobular electric field strength between the two ctDCS montages for all ten subjects.

### Effects of ctDCS on the balance and gait related clinical measures

The results from the clinical assessment of the TMWT, TUG, and the BBS are presented in the Supplementary Materials that showed a small improvement in the TMWT (MCID: 0.10 m/s), TUG (MCID: 8 s), and BBS score (MCID: 12.5 points) for both the ctDCS montages. These indicated an improved walking speed that can be helpful for community ambulation [45]; however, the improvements were similar across the ctDCS montages (details provided in the supplementary materials).

## Discussion

In this feasibility study, we investigated the effects of two different ctDCS montages on overground gait parameters. Data on subjective feedback (the questionnaire provided in the supplementary materials) suggests that post-stroke patients in this study could tolerate the ctDCS with 3.14cm^2^ disc gel electrodes at 2 mA direct current. The ctDCS montages were computationally optimized for targeting the dentate nuclei (in dentate ctDCS) and the leg representations (in leg ctDCS) in the cerebellum. Both the ctDCS montages resulted in higher than 0.1 V/m electric field strength at non-targeted cerebellar regions, as shown in Fig. 3. In fact, we found it challenging to avoid affecting the dentate nucleus, the largest of the deep cerebellar nuclei, when targeting leg lobules VIIb-IX. Such a spill-over effect on the dentate nucleus and non-cerebellar brain regions was found for conventional ctDCS montages, including Celnik’s ctDCS montage with one electrode on one cerebellar hemisphere and the other electrode on the ipsilateral buccinators muscle [22]. The ctDCS effects are primarily studied based on the impact of the Purkinje cell [41] under the quasi-uniform assumption that local polarization effect is proportional to the local electric field strength [50]; however, the local electric field effects on the dentate nuclei should also be considered [51]. Such cerebellar sub-structure specific investigation of ctDCS electric field is crucial to decrease the barriers to replicability [52].

Motor skill acquisition and retention can have different processes, viz., ctDCS of the posterior cerebellum (related to the complexity of the motor performance [19]) using Celnik’s montage facilitated a reduction of movement errors during skill acquisition [53]. Here, Celnik’s ctDCS montage [24] primarily affected the posterior cerebellum related to the "cognitive" cortico-striatal loop [54], including the lobules Crus I/II, VIIb, VIII, and IX of the targeted cerebellar hemisphere [22]. In contrast, ctDCS optimized for the anterior cerebellum, related to the motor network [19], can be postulated to facilitate the development of "motor memory" [55, 56] or skill retention after extensive practice where these effects can be comparable to M1 tDCS effect that also facilitated increased retention of the new skill [24]. Here, M1 and the premotor cortices are related to the implementation of the motor commands that is facilitated via cerebellar projections to M1 as well as premotor and other frontal regions [57]. After extensive practice, the "motor" loop gets involved in developing "motor memory" [54] that consists of motor, premotor, somatosensory, supplementary motor areas, and anterior cerebellum that are involved in performance optimization. Therefore, cerebellar sub-structure specific optimization of ctDCS electric field related to various stages of motor skill acquisition and retention is crucial.

Our prior work [31] showed that anodal ctDCS of the anterior lobe of the cerebellum during visuomotor learning of myoelectric visual pursuit using electromyogram (EMG) from gastrocnemius muscle resulted in a statistically significant (p < 0.05) decrease in the reaction time ("motor" loop effect?) post-intervention than baseline when compared to anodal ctDCS of the posterior lobe of the cerebellum as well as anodal ctDCS of combined anterior and posterior lobes of the cerebellum; however, only anodal ctDCS of combined anterior and posterior lobes of cerebellum resulted in a significant decrease in root mean square error ("cognitive" loop effect?) post-intervention than in the baseline. Here, deep ctDCS of dentate nuclei resulted in electric field distribution over combined anterior and posterior lobes of the cerebellum that was found in our prior work using computational modeling [23]. So, in the current study, we targeted the dentate nuclei in addition to either combined anterior and posterior lobes of the cerebellum (in dentate ctDCS) or the leg lobules VIIb-IX in the posterior cerebellum (in leg ctDCS). We found opposite effects on the 'Step Time Affected Leg' and the '%Stance Time Unaffected Leg' where dentate ctDCS decreased them both, which is postulated to be due to the connection of the anterior lobe with the movement frequency [19]. In prior work [58], Celnik’s ctDCS montage, primarily affecting the posterior cerebellum, affected the adaptation rate of spatial but not temporal elements of gait. The opposite effects of anterior and posterior lobes of the cerebellum can be investigated by focally targeting with CLOS [22] in future studies.

Our feasibility study was not adequately powered with the probability of detecting ctDCS montage specific effects of clinical importance [59], and both the ctDCS montages (dentate ctDCS and leg ctDCS) were found to have a small effect on the clinical results from the TMWT, BBS, TUG (details in the supplementary materials). However, quantitative gait analysis showed ctDCS montage specific effects for the 'Step Time Affected Leg' (p = 0.0257) and '%Stance Time Unaffected Leg' (p = 0.0376) using a two-sided Wilcoxon rank-sum test at 5% significance level, as shown in Fig. 4. These changes can be attributed to the ctDCS electric field strength since individual changes in the quantitative gait parameters across both the montages were found to be correlated to the mean electric field strength in the lobules based on PLSR analysis (see Fig. 5). Here, the loadings of the latent variables found from the PLSR analysis (see Fig. 6) for the response variables (percent normalized change in gait parameters) and the predictor variables (mean lobular electric field strength) showed that the mean electric field strength at the posterior cerebellar lobules, Vermis VIIIb, Ipsi-lesional IX, Vermis IX, Ipsi-lesional X, were primarily related to the 'Step Time Affected Leg' and '%Stance Time Unaffected Leg.' Both the ctDCS montages used ipsilesional cathode where 'Step Time Affected Leg' (p = 0.0257) and '%Stance Time Unaffected Leg' (p = 0.0376) showed significant montage specific effects at 5% significance level, which may be related to ctDCS facilitating cerebellum in providing a learned timing signal [32]. The ipsilesional cathode effects may be related to the CBI, where prior work in healthy subjects showed robust effects of cathodal ctDCS after-effects on CBI [60]. Such effects of CBI on the contra-lesional leg M1 can be compared with the results from Tahtis et al. [14] where bi-cephalic tDCS with cathode placed over the contra-lesional leg motor cortex improved the gait functionality of post-stroke patients that was postulated due to reduction of the excitability of the contra-lesional leg motor cortex. Also, the tDCS effect on the lobule VIIIb and X is postulated to be related to the moderate effect of ctDCS on the 'Gait Stability Ratio' (p = 0.0569), which has been shown to be an indicator of balance during walking [35]. This postulate is based on prior works that showed that the posterior vermis is related to the performance on tandem walking [61], lobule X was found essential in the vestibular system [62], and the motor and somatosensory activation were linked to the lobule VIIIb [63]. Also, moderate effects were found for the 'Normalised Step length Affected side' (p = 0.1) that may be related to the ctDCS effects on the lobules VIIIb and IX [27]. These posterior cerebellar lobules VIIIb, IX, and X were affected by comparable mean electric field strength (> 0.1 V/m) in both ctDCS montages, as shown in Fig. 6c, which may have led to similar balance and gait-related behavioral outcomes across the two ctDCS montages (TMWT, BBS, TUG results in the supplementary materials).

Clinical literature shows a crucial role of the cerebellum in coordinating voluntary movements (e.g., walking) and maintaining a biped balance [64]. In this feasibility study, the cerebellum was intact in our subjects so that the contra-lesional anodal ctDCS was performed to alleviate deficits in the motor network in the cerebrum. Prior work on the random-effects modeling of the cumulative effect size by Oldrati and Schutter [65] showed that both anodal and cathodal ctDCS were effective in changing motor- and cognitive-related behavioral performance in healthy volunteers. Here, the polarity of the ctDCS was not predictive of the direction of the behavioral changes in healthy volunteers [65]. We have found robust effects of the cathodal ctDCS on CBI [60]; however, the clinical applicability of ctDCS in improving the functional gait ability remained unexplored. Due to a diversity of ideas on cerebellar involvement in the movement and the inter-subject variability in the ctDCS effects [19], we proposed a multivariate brain (electric field strength)—behavior (movement measures) regression modeling [23]. In this feasibility study, we found that PLSR analysis can be an effective technique for multivariate regression modeling to understand the relation between the electric field distribution and the behavioral effects where PLSR results can also be generalized (i.e., to create a random effect model) in the future using inferential analytical approach [49] for ctDCS dosing using a larger dataset.

In the current study, we aimed for feasibility testing of multivariate regression analysis to test an association between the lobular mean electric field strength in a single ctDCS session and the quantitative effects on gait parameters in chronic stroke. Here, ctDCS montages were not optimized with an individualized lesioned head model that limited any subject-specific inferences using the PLSR analysis, considering that the post-stroke participants had heterogeneous lesion conditions in the cerebrum that were not modeled during the electric field analysis. In this study, the electric field strength was mostly limited to the cerebellum (< 0.1 V/m in the non-cerebellar brain—see Fig. 3), and the cerebellum was intact in all the post-stroke subjects selected for this study (confirmed with computerized tomography scan). Heterogeneous lesion locations in the cerebrum need further investigation vis-à-vis non-responders, where multi-block or multi-table PLS can integrate one or more of these classed in a common analysis [49]. In the future, we also plan to segregate the extended pool of post-stroke participants involving more post-stroke participants from both genders based on the behavioral measures in addition to portable neuroimaging of the ctDCS response in the cerebrum [66] for such multi-table PLSR analysis.

Our preliminary findings in this feasibility study are encouraging; however, this study had certain limitations. The main limitation is the lack of a sham ctDCS group that is necessary to test clinically meaningful hypotheses. Also, our statistical analysis in this feasibility study is mainly descriptive [59] due to a small sample size of chronic post-stroke participants with heterogeneous conditions. The low statistical power has a reduced chance of detecting a true montage-specific effect [67], so this study can be considered only a proof-of-concept, i.e., not adequately powered with placebo control for clinical validation. Moreover, a 2–3 days’ washout period was provided between ctDCS sessions, and the carry-over effects were not evaluated based on neurophysiological testing in this study. In addition, the electrode locations were not optimized with individual MRI, although montages were optimized based on the subject's age-specific head model without brain lesions. Furthermore, the convenience sampling in this study was biased since all of our participants were male hemiplegics, with four being left hemiplegic and the remaining six right hemiplegics. Nevertheless, randomized order ensured baseline equivalence between the two groups.

## Conclusion

Our feasibility study indicated an association between the lobular mean electric field strength and the quantitative effects on gait parameters in chronic stroke based on PLSR analysis. Here, the quantitative gait parameters across both the montages were found to be correlated to the mean lobular electric field strength following a single ctDCS session, which can be considered a first step towards understanding the underlying mechanisms of ctDCS. Our PLSR results can be generalized (i.e., to create a random effect model) in the future using the inferential analytical approach for dosing ctDCS, including identification of non-responders, for planning long-term clinical intervention.

## Supplementary Information

**Additional file 1: Figure S1.** Clinical Gait Parameters before (Pre) and after (Post) the application of leg ctDCS. (a) Ten-Meter Walk Test (b) Timed-Up and Go Test (c) Berg Balance Scale score. Note: Ten-Meter Walk Test MCID Value: 0.10 m/s [1]; Timed-Up and Go Test MCID Value: 8 s [1]; BBS Score MCID Value: 12.5 points [2]. **Figure S2.** Clinical Gait Parameters before (Pre) and after (Post) the application of dentate ctDCS. (a) Ten-Meter Walk Test (b) Timed-Up and Go Test (c) Berg Balance Scale score. Note: Ten-Meter Walk Test MCID Value: 0.10 m/s [1]; Timed-Up and Go Test MCID Value: 8 s [1]; BBS Score MCID Value: 12.5 points [2]. **Figure S3.** Spatiotemporal gait parameters during overground walking before (Pre) and after (Post) the application of leg ctDCS. (a) Normalised Stride Length for Affected leg (b) Normalised Stride Length for Unaffected Leg (c) Walk Ratio for Affected Leg (d) Walk Ratio for Unaffected Leg (e) Gait Stability Ratio (f) Symmetry Index (g) Stride Time for Affected Leg (h) Stride Time for Unaffected Leg (i) Step Time for Affected Leg (j) Step Time for Unaffected Leg (k) %Stance Time for Affected Leg (l) %Stance Time for Unaffected Leg (m) %Swing Time for Affected Leg (n) %Swing Time for Unaffected Leg (o) % Single Support Time for Affected Leg (p) % Single Support Time for Unaffected Leg (q) Cadence. **Figure S4.** Spatiotemporal gait parameters during overground walking before (Pre) and after (Post) the application of dentate ctDCS. (a) Normalised Stride Length for Affected leg (b) Normalised Stride Length for Unaffected Leg (c) Walk Ratio for Affected Leg (d) Walk Ratio for Unaffected Leg (e) Gait Stability Ratio (f) Symmetry Index (g) Stride Time for Affected Leg (h) Stride Time for Unaffected Leg (i) Step Time for Affected Leg (j) Step Time for Unaffected Leg (k) %Stance Time for Affected Leg (l) %Stance Time for Unaffected Leg (m) %Swing Time for Affected Leg (n) %Swing Time for Unaffected Leg (o) % Single Support Time for Affected Leg (p) % Single Support Time for Unaffected Leg (q) Cadence.

## Data Availability

Supporting data is available from the first author, Dhaval Solanki.
