# Supplementary material for: Investigating the feasibility of cerebellar transcranial direct current stimulation to facilitate post-stroke overground gait performance in chronic stroke: a partial least-squares regression approach
Source: J Neuroeng Rehabil. 2021 Jan 28;18:18. doi: 10.1186/s12984-021-00817-3 (PMC7842063; doi:10.1186/s12984-021-00817-3)
Supplement: Supplementary file 1 — Additional file 1: Figure S1. Clinical Gait Parameters before (Pre) and after (Post) the application of leg ctDCS. (a) Ten-Meter Walk Test (b) Timed-Up and Go Test (c) Berg Balance Scale score. Note: Ten-Meter Walk Test MCID Value: 0.10 m/s [1]; Timed-Up and Go Test MCID Value: 8 s [1]; BBS Score MCID Value: 12.5 points [2]. Figure S2. Clinical Gait Parameters before (Pre) and after (Post) the application of dentate ctDCS. (a) Ten-Meter Walk Test (b) Timed-Up and Go Test (c) Berg Balance Scale score. Note: Ten-Meter Walk Test MCID Value: 0.10 m/s [1]; Timed-Up and Go Test MCID Value: 8 s [1]; BBS Score MCID Value: 12.5 points [2]. Figure S3. Spatiotemporal gait parameters during overground walking before (Pre) and after (Post) the application of leg ctDCS. (a) Normalised Stride Length for Affected leg (b) Normalised Stride Length for Unaffected Leg (c) Walk Ratio for Affected Leg (d) Walk Ratio for Unaffected Leg (e) Gait Stability Ratio (f) Symmetry Index (g) Stride Time for Affected Leg (h) Stride Time for Unaffected Leg (i) Step Time for Affected Leg (j) Step Time for Unaffected Leg (k) %Stance Time for Affected Leg (l) %Stance Time for Unaffected Leg (m) %Swing Time for Affected Leg (n) %Swing Time for Unaffected Leg (o) % Single Support Time for Affected Leg (p) % Single Support Time for Unaffected Leg (q) Cadence. Figure S4. Spatiotemporal gait parameters during overground walking before (Pre) and after (Post) the application of dentate ctDCS. (a) Normalised Stride Length for Affected leg (b) Normalised Stride Length for Unaffected Leg (c) Walk Ratio for Affected Leg (d) Walk Ratio for Unaffected Leg (e) Gait Stability Ratio (f) Symmetry Index (g) Stride Time for Affected Leg (h) Stride Time for Unaffected Leg (i) Step Time for Affected Leg (j) Step Time for Unaffected Leg (k) %Stance Time for Affected Leg (l) %Stance Time for Unaffected Leg (m) %Swing Time for Affected Leg (n) %Swing Time for Unaffected Leg (o) % Single Support Time for Affected Leg (p) % Sin [file 12984_2021_817_MOESM1_ESM.pdf]

Additional Materials

This supplementary file comprises of 1) Clinical Gait Measures before (Pre) and after (Post) the application of ctDCS as well as minimal clinically important differences (MCID) between the Pre and Post intervention, 2) Spatiotemporal Gait Parameters in the case of leg ctDCS at lobules VIIb-IX, 3) Spatiotemporal Gait Parameters in the case of dentate ctDCS, and 4) User Feedback Questionnaires.

1. Clinical Gait Measures Pre and Post application of cerebellar tDCS

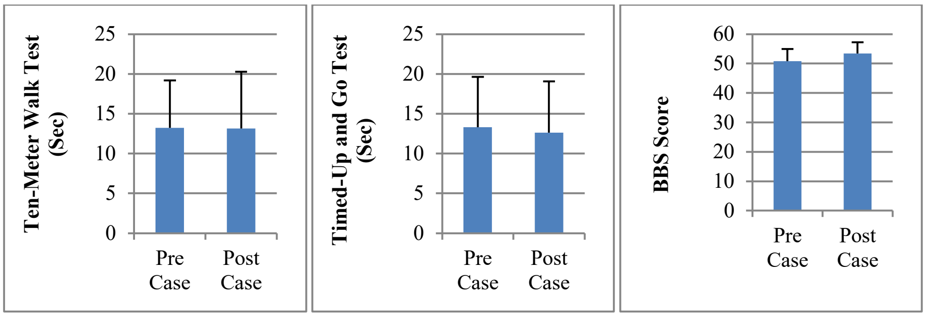

(a) (b) (c)

Figure 1. Clinical Gait Parameters before (Pre) and after (Post) the application of leg ctDCS. (a) Ten-Meter Walk Test (b) Timed-Up and Go Test (c) Berg Balance Scale score. Note: Ten-Meter Walk Test MCID Value: 0.10 m/s [1]; Timed-Up and Go Test MCID Value: 8 Sec [1]; BBS Score MCID Value: 12.5 points [2].

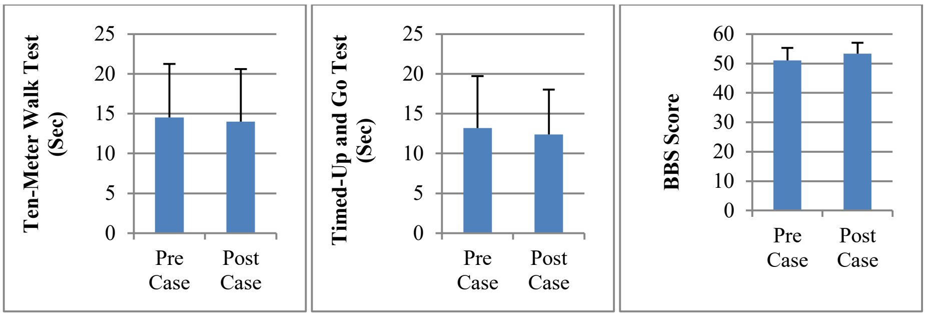

(a) (b) (c)

Figure 2. Clinical Gait Parameters before (Pre) and after (Post) the application of dentate ctDCS. (a) Ten-Meter Walk Test (b) Timed-Up and Go Test (c) Berg Balance Scale score. Note: Ten-Meter Walk Test MCID Value: 0.10 m/s [1]; Timed-Up and Go Test MCID Value: 8 Sec [1]; BBS Score MCID Value: 12.5 points [2]

2. Spatiotemporal Gait Parameters before and after leg ctDCS

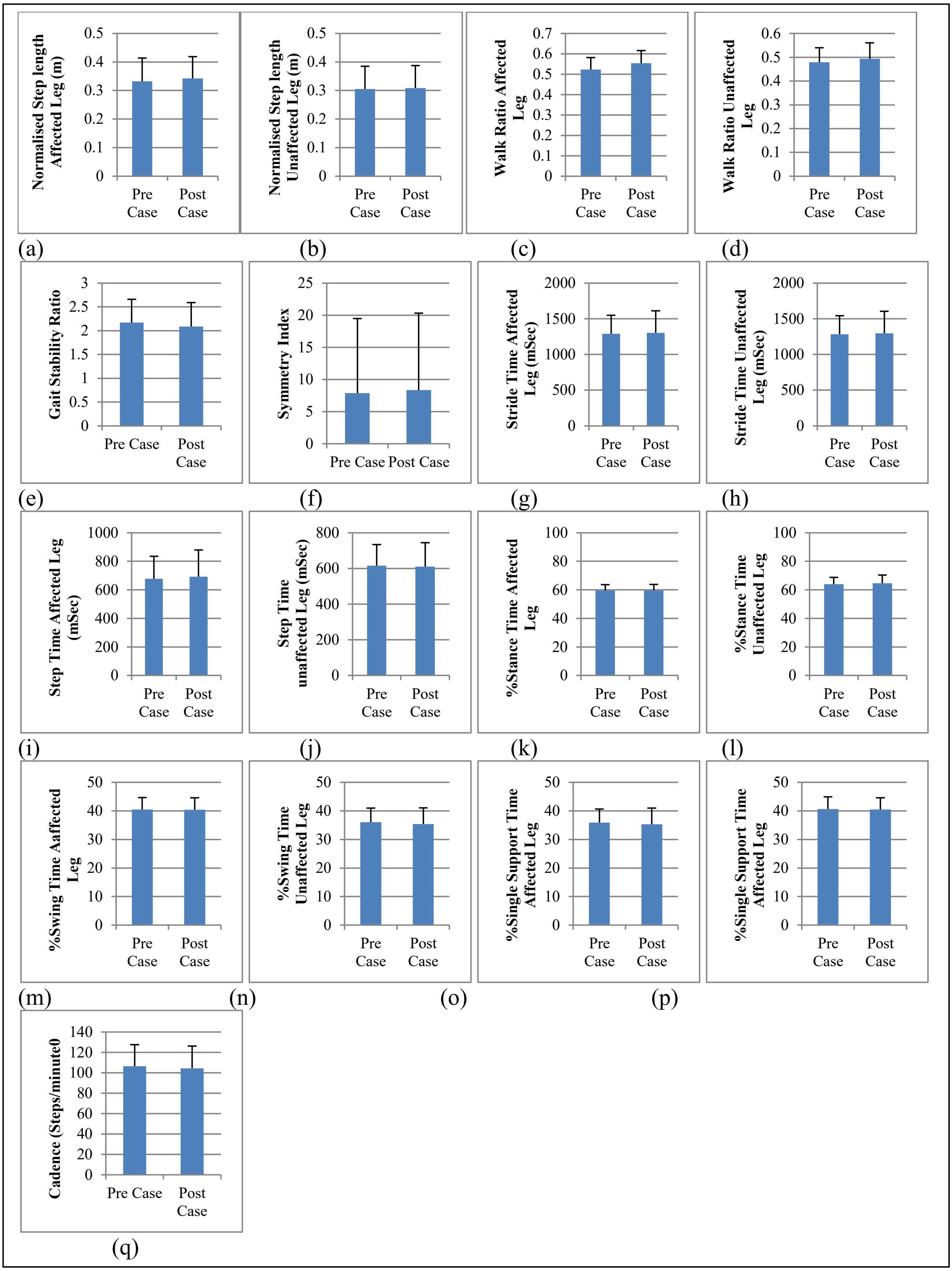

Figure 3: Spatiotemporal gait parameters during overground walking before (Pre) and after (Post) the application of leg ctDCS. (a) Normalised Stride Length for Affected leg (b) Normalised Stride Length for Unaffected Leg (c) Walk Ratio for Affected Leg (d) Walk Ratio for Unaffected Leg (e) Gait Stability Ratio (f) Symmetry Index (g) Stride Time for Affected Leg (h) Stride Time for Unaffected Leg (i) Step Time for Affected Leg (j) Step Time for Unaffected Leg (k) %Stance Time for Affected Leg (l) %Stance Time for Unaffected Leg (m) %Swing Time for Affected Leg (n) %Swing Time for Unaffected Leg (o) % Single Support Time for Affected Leg (p) % Single Support Time for Unaffected Leg (q) Cadence

3. Spatiotemporal Gait Parameters before and after dentate ctDCS

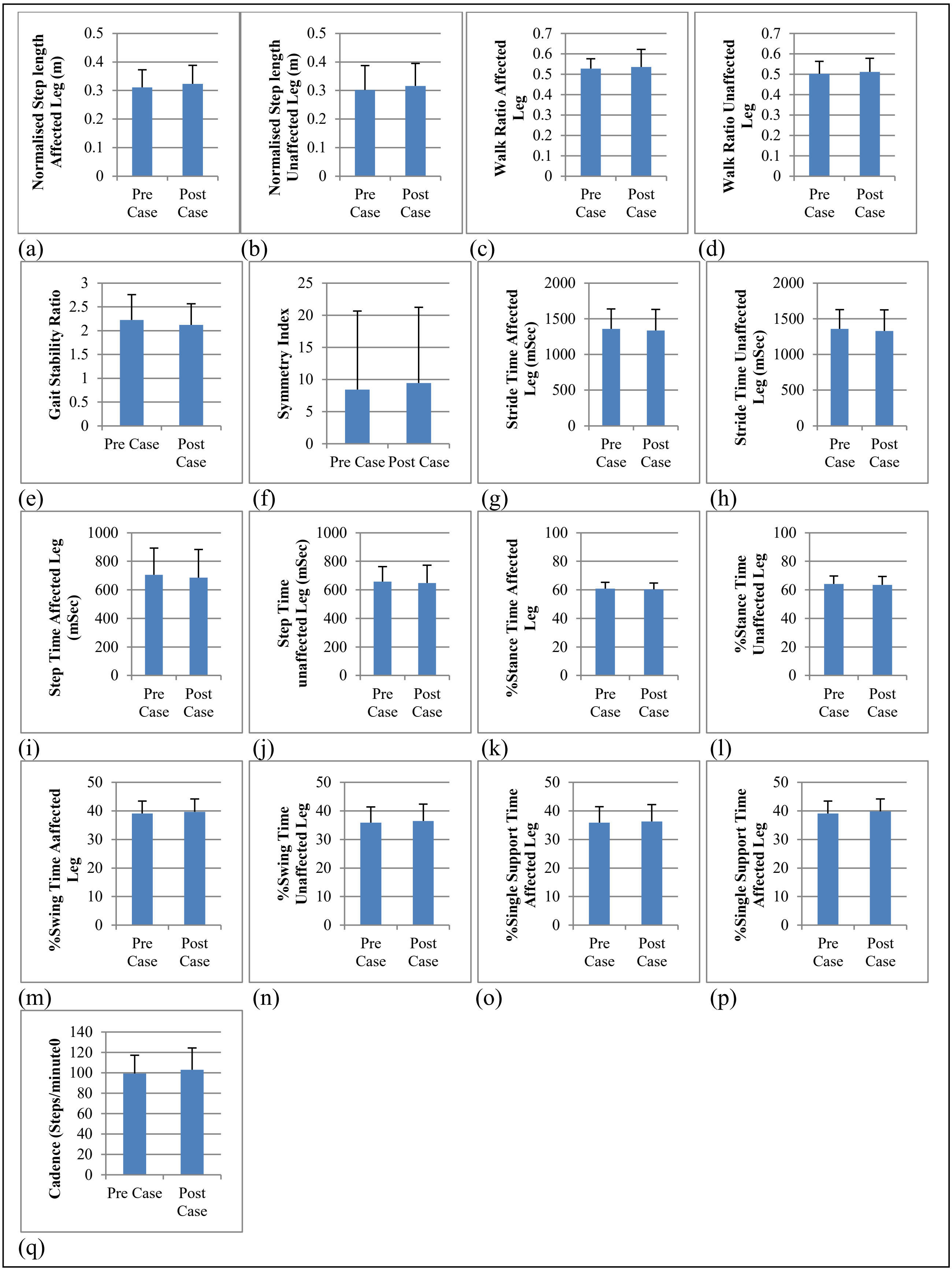

Figure 4: Spatiotemporal gait parameters during overground walking before (Pre) and after (Post) the application of dentate ctDCS. (a) Normalised Stride Length for Affected leg (b) Normalised Stride Length for Unaffected Leg (c) Walk Ratio for Affected Leg (d) Walk Ratio for Unaffected Leg (e) Gait Stability Ratio (f) Symmetry Index (g) Stride Time for Affected Leg (h) Stride Time for Unaffected Leg (i) Step Time for Affected Leg (j) Step Time for Unaffected Leg (k) %Stance Time for Affected Leg (l) %Stance Time for Unaffected Leg (m) %Swing Time for Affected Leg (n) %Swing Time for Unaffected Leg (o) % Single Support Time for Affected Leg (p) % Single Support Time for Unaffected Leg (q) Cadence

4. User Feedback (Questionnaires)

Before investigating the possibility of using ctDCS in gait rehabilitation of post-stroke patients, it is necessary to understand whether the application of ctDCS is acceptable to the target population. To understand the patients' perception, we collected feedback from the post-stroke participants based on their feedback prior to (Pre_{tDCS}), during ($Active_{tDCS}$) and post ($Post_{tDCS}$) application of ctDCS. This was conducted in the form of a small survey comprising of three questions (Q1-Q3, Table 1) adopted from the User Sustainability Evaluation Questionnaire [3]. After the cap with the electrodes and the portable tDCS device was placed on the participant's head, we obtained the feedback on the Pre_{tDCS} experience of the participant to understand whether the patient was comfortable with the cap (Q1). All of the participants (except P8 and P11) expressed that they were comfortable wearing the cap. The Pre_{tDCS} session was followed by the $Active_{tDCS}$ in which the participant was offered ctDCS. None of the participants expressed any discomfort with the current stimulation during the $Active_{tDCS}$ (Q2), though most of them reported that they could feel minor tingling sensation on the scalp for few seconds that was tolerable. Following the application of ctDCS, the user feedback based on their experience was obtained, i.e., $Post_{tDCS}$, that revealed that none of the participants had any adverse effect, such as burning sensation, nausea, headache. Also, no skin reddening (at the location of electrodes) of the scalp was found (Q3).

Table 1: Questionnaires to understand acceptability of ctDCS by post-stroke patients.

ID	Question
Q1	Did you enjoy your experience with the setup?
Q2	Did you feel comfortable with the setup?
Q3	Did you experience any side effects, e.g., sensation of tissue burning, nausea, headache?

References

[1] Mazzoleni, Stefano, et al. "Robot-assisted end-effector-based gait training in chronic stroke patients: A multicentric uncontrolled observational retrospective clinical study." NeuroRehabilitation 40.4 (2017): 483-492.

[2] Song, Min-Jeong, Jae-Hyoung Lee, and Won-Seob Shin. "Minimal Clinically Important Difference of Berg Balance Scale scores in people with acute stroke." Physical Therapy Rehabilitation Science 7.3 (2018): 102-108.

[3] Gil-Gómez, J. A., Manzano-Hernández, P., Albiol-Pérez, S., Aula-Valero, C., Gil-Gómez, H., & Lozano-Quilis, J. A. (2017). USEQ: a short questionnaire for satisfaction evaluation of virtual rehabilitation systems. Sensors, 17(7), 1589.
